# A Case Report of Probable Paliperidone ER-Induced Serotonin Syndrome in a 17-Year-Old Taiwanese Female With New Onset Psychosis

**DOI:** 10.1097/MD.0000000000002930

**Published:** 2016-03-07

**Authors:** Chung-Hao Yang, Kai-Dih Juang, Po-Han Chou, Chin-Hong Chan

**Affiliations:** From the Department of Psychiatry (C-HY, K-DJ, P-HC), Taichung Veterans Hospital, Taichung; and Department of Psychiatry (C-HC), Conde S. Januário General Hospital, Macau, China.

## Abstract

A 17-year-old female with new-onset psychosis was treated with paliperidone. After increasing the paliperidone dose to 12 mg per day the patient developed a series of side effects; Tachycardia (140 bpm), severe drooling, restlessness, diaphoresis, whole-body tremor, inducible foot clonus, predominant lower limbs rigidity, bilateral pupil dilation, increased bowel sounds with watery diarrhea, and muscle hypertonicity. The symptoms subsided after stopping the paliperidone, and recurred after resuming paliperidone 9 mg per day.

To our knowledge, this is the first case of a very clear and close relationship between the symptoms of serotonin syndrome and the use of paliperidone. We have to cautiously consider the diagnosis of serotonin syndrome in potential cases.

## INTRODUCTION

Serotonin syndrome (SS) is a potentially life-threatening adverse effect of serotonergic drugs.^1^ The classic clinical triad of SS includes mental status changes, autonomic hyperactivity, and neuromuscular abnormalities. Neurologic examination may reveal intermittent tremor or myoclonus, and hyperreflexia.^1,2^ We describe “A case of paliperidone-induced SS is described within a 17-year-old female showing new-onset psychosis.”

## CASE DESCRIPTION

A 17-year-old female was admitted to the pediatric ward due to acute psychosis including disorganized behavior and auditory hallucination for the duration of 2 weeks.

Comprehensive evaluation including drug abuse test by standard urine immunoassay revealed no previous physical illness or substance exposure (nicotine, alcohol, betel nut, and illegal substances). Cerebrospinal fluid was obtained through lumbar puncture for the examinations of infectious agents; however findings were inconclusive. A psychiatrist was consulted and paliperidone-ER oral form 9 mg per day was prescribed 1 week after admission. She was discharged the next day; however, she had poor drug compliance after discharge. She was admitted to the acute psychiatric ward 10 days later due to persistent psychosis. Medication prescribed during admission included; paliperidone-ER (9 mg per day) and estazolam (2 mg per night). Tachycardia (131 beats per minute) was noted on the second day. The dosage of paliperidone was increased to 12 mg on the fifth day of admission due to the lack of improvement in psychosis. Lithium (900 mg per day) was also prescribed as an augmentation therapy. Further increase in tachycardia was noted (140 bpm). In addition, her body temperature rose to 37.7 °C. Furthermore, the patient again developed various symptoms the following day including severe drooling, restlessness, diaphoresis, whole-body tremor, inducible foot clonus, bilateral pupil dilation, increased bowel sounds with watery diarrhea, lower limbs predominant joint rigidity, and muscle hypertonicity. Following this she became extremely agitated and confused. These symptoms did not subside after the injections of anticholinergic agents or lorazepam: SS was highly suspected. Thus, the antipsychotic medication was changed from paliperidone-ER to quetiapine (200 mg per day) with supportive treatment (glucose saline IV fluid 1500 cc per day).

It was suspected that the SS might have been caused by the combination of lithium and paliperidone-ER; therefore, the use of lithium was also stopped. During the next 3 days, her heart rate dropped below 100 bpm, and her mental status, autonomic hyperactivity, and neuromuscular abnormalities also improved. However, her psychotic symptoms had recurred. In an attempt to address the situation quetiapine was changed back to paliperidone-ER (9 mg per/day). However, the previous adverse effects returned after the change in medication (list the adverse effects here). Inducible foot clonus, diaphoresis, predominant lower limbs rigidity, muscle hypertonicity, dilated pupil, and hypersalivation occurred the next day. She became agitated and confused again. Her vital signs suggested mild elated in body temperature (maximum 37.3 °C), tachycardia (maximum 146 bpm), relatively stable blood pressure (108/86–123/93 mm Hg). Her blood creatine kinase level was 56 U/L, and WBC was 8800/CUMM. Following this, the medication was changed back to Quetiapine. The patient regained clear consciousness and showed complete improvement in autonomic and neurological symptoms within 3 days. She was then treated with quetiapine (200 mg per day) and was discharged in a stable condition without any obvious signs of psychosis 20 days later.

After given fully informed, the permission for this case report from our patient and her parents are obtained. They have agreed to participate in this case report. On the other hand, our case report does not need ethical approval from ethics committee or institutional review board.

## DISCUSSION

It was reported that the symptoms found in this case study resemble that of those found in the Sternback criteria of SS (mental status changes, agitation, myoclonus, hyperreflexia, diaphoresis, shivering, tremor, diarrhea).^3^ This is the first case report of SS induced by paliperidone. Paliperidone is an atypical antipsychotic with serotonin 5-HT_2a_, 5-HT_7_, and D_2_ receptor antagonism. Paliperidone is not considered a serotonergic agent, and no paliperidone-related SS has been previously reported. It is suggested that Paliperidone could be a “probable” cause of SS in this case as revealed from the assessment of Naranjo Nomogram.^4^ As lithium has some serotonergic properties, we cannot rule out the effect by lithium, although we stopped the use of lithium when resuming paliperidone.

However, several other diagnoses, including neuroleptic malignant syndrome (NMS), anticholinergic toxidrome, sympathomimetics symptoms, and anti-NMDAR encephalitis, should be taken into consideration. NMS is characterized by fever, muscular rigidity, altered mental status, and autonomic instability .^5^ There have been 5 case reports of NMS caused by paliperidone.^6–10^ Compared with case reports from Han et al, Nayak et al, and Ozdemir et al, our case did not have hyperthermia over 38°C or large elevation of creatine kinase in serum (at least 2000 IU/L in their cases). However, our case still has possibility to be a mild form of NMS. The case mentioned by Duggal only had low grade fever and relatively low elation in creatine kinase. Unlike other case reports mentioned, our case did not increase muscle tone in all 4 limbs, instead only predomninant in lower limbs. The presence of mydriasis, hyperactive bowel sounds, inducible clonus, increasing lower limbs muscle tone in our case are also not characteristics of NMS and not reported in other previous cases. It makes it unlikely this syndrome represented NMS rather than SS. Mydriasis, tachycardia, low-grade fever, and altered mental status in our case might appear similar to anticholinergic toxidrome. However, the patient had not taken anticholinergic agents before the symptoms appeared. Sialorrhea, hyperactive bowel sounds, and hyperreflexia make anticholinergic “toxidrome” less likely. However, peripheral signs of intact cholinergic function cannot rule out central anticholinergia completely. Patients who have taken amphetamines or cocaine commonly present with tachycardia, hypertension, agitation, diaphoresis, and mydriasis, and may have psychotic behavior. The patient had no history of illicit drug or alcohol abuse and the urine immunoassay test also failed to identify any dangerous drugs. Moreover in our case neither the history of the patient nor the course of the disease supported the diagnosis Anti-NMDAR encephalitis.

The exact mechanism remains unknown. Paliperidone is a potent antagonist of the 5-HT_7_ receptor, and 5-HT_7_ inhibition produces the same behavioral effects as antidepressants that increase 5-HT transmission.^11^ One explanation is that, like the 5-HT_1a_ receptors, with which they share functional and morphological characteristics, the 5-HT_7_ receptors may have opposite effects on serotonergic transmission. Perhaps this is the possible cause of paliperidone-induced SS.

## CONCLUSIONS

This case report demonstrates a possible example of paliperidone induced SS, which can be reversed by discontinuation of the drug. Clinician should cautiously consider the diagnosis of serotonin syndrome in potential cases and stop the use of antipsychotics.
